# PSpice modeling of cervical and site-focused vagus nerve ultrasonic stimulation for reduced tumor necrosis factor-α production

**DOI:** 10.1038/s41598-022-25944-2

**Published:** 2022-12-12

**Authors:** Sleiman R. Ghorayeb, Bryan Hirsch

**Affiliations:** 1 Department of Bioengineering, Fred DeMatteis School of Engineering and Applied Science, Hofstra University, Hempstead, NY 11549 USA; 2 Ultrasound Research Laboratory, Hofstra University, Hempstead, NY 11549 USA; 3 Departments of Radiology and Molecular Medicine, Donald and Barbara Zucker School of Medicine at Hofstra/Northwell, Hofstra University, Hempstead, NY 11549 USA

**Keywords:** Network models, Neural circuits, Biomedical engineering, Electrical and electronic engineering

## Abstract

Clinical ultrasound is widely used as a diagnostic and therapeutic tool. Recently, it has been used to perform neuromodulation to treat diverse effects, including inflammation reduction through the vagus nerve. Although the mechanism by which ultrasound propagates through tissue for diagnostic purposes has been established, there is not a complete understanding of how it interacts with neurons to elicit excitation and inhibit inflammation. This work presents a novel technique based on a well-established electrical engineering tool, PSpice, to model cervical and site-focused vagus nerve ultrasonic stimulation to understand its capability in reducing tumor necrosis factor-α (TNF-α) production in the spleen. Transmission line theory is utilized as the basis for the different tissue layers. The models supported the hypothesis that site-focused stimulation has the advantage to decrease undesired efferent effects that would otherwise occur with cervical stimulation. Two different acoustic pressures, 0.25 and 0.83 MPa, were simulated for theoretical efficacy and safety based on previous experimental work conducted by others. The 0.25 MPa simulation was ideal for neurostimulation and reduction of TNF-α, while 0.83 MPa resulted in much higher intensity levels that will most likely induce additional inflammation.

## Introduction

High frequency ultrasound has been utilized in diagnostic imaging for decades. Additionally, low frequency therapeutic ultrasound has numerous applications ranging from deep tissue healing to surgical scalpels. More recently, ultrasound was employed remedially where the acoustic waves interact with viscera to treat an ailment, eliciting a specific response. One such response involves the immune system, targeting the vagus nerve to decrease systemic inflammation. The vagus nerve being the tenth cranial nerve and the largest nerve in the body.

Ultrasonic vagus nerve stimulation (VNS) used to reduce inflammation is largely associated with autoimmune diseases, including rheumatoid arthritis, colitis, inflammatory bowel disease (IBD), and Crohn’s disease, among others. Studies such as the ones conducted by Cotero et al.^1^ and Juan et al.^2^ discuss organ specific and cervical stimulation of the vagus nerve respectively, to initiate the cholinergic anti-inflammatory pathway. The cervical stimulation of the vagus nerve pathway is composed of the vagus nerve, the splenic nerve, and ultimately the spleen, where immune cells, such as T-cells, are located^3^. In Cotero’s site-focused pulsed ultrasound, the ultrasonic transducer was positioned over the spleen, as indicated by the cholinergic anti-inflammatory pathway. The shift from the cervical to site-focused position reduces the chance of undesirable efferent effects associated with cervical stimulation.

To target the spleen with cervical VNS, a signal is transmitted from the neck region of the vagus nerve to the celiac plexus, from where the splenic nerve bifurcates. The splenic nerve transmits the signals from the celiac plexus to the spleen, where 90% of TNF-α, a major inflammatory cytokine, is produced and released systemically^4^. The peripheral splenic nerve fibers terminate in T-cell rich areas, as indicated by the arrow in Supplementary Fig. S1. Splenic nerve fibers fall under the class of adrenergic nerves. Adrenergic nerves release norepinephrine as its neurotransmitter compared to the more common cholinergic nerves that release acetylcholine. Splenic nerve stimulation increases norepinephrine in these T-cell rich regions where the nerve fibers form synaptic-like structures with the immune cells. This allows for an intimate interaction between the efferent vagus nerve and the proposed mechanism for TNF-α inhibition. The norepinephrine binds to β_2_-andrenergic receptors located on the surface of T-cells^4^. A proposed mechanism for acetylcholine production by T-cells utilizes choline acetyltransferase (ChAT). ChAT activity is regulated by phosphorylation via protein kinase C (PKC), Ca^2+^/calmodulin kinase II, and protein kinase A (PKA), but the specific mechanism is not known^4^. Acetylcholine is released by T-cells and binds to α7 nicotinic acetylcholine receptors (α7nAChR) on the surface of macrophages, inhibiting TNF-α production that occurs within them.

The mechanism with which ultrasound waves stimulate and interact with neurons and biological tissue is fundamental for the reduction of inflammation. One proposed mechanism is the neuronal intramembrane cavitation excitation (NICE) model^5^. In this model, ultrasonic waves interact with neuronal lipid bilayers. The negative and positive pressure from acoustic waves pull apart and push together the lipid bilayer, respectively. This motion introduces cavitation, nanobubbles, in between the lipid bilayer monolayers, formed of a dissolved gas that expands and contracts in an oscillating manner. These nanobubbles are generated at intensity levels greater than 0.1 W/cm^2^ and frequencies of approximately 1 MHz^6^. The introduction of cavitation alters regional membrane curvature and in turn membrane capacitance. The change in capacitance by intramembrane cavitation, as a result of acoustic waves, induces an alternating hyperpolarizing current, ultimately leading to an action potential^5^. The resultant action potential follows the mechanism described by Hodgkin and Huxley based on ion concentration flow^7^.

This paper serves to illustrate the efficacy of ultrasonic neurostimulation, as well as the reduction in efferent vagus nerve pathway excitation by way of site-focused treatment. The ultrasonic wave propagation through the vagus nerve was analyzed with the use of a circuit simulation program PSpice (Orcad™; Hillsboro, OR). A similar approach was investigated by Ghorayeb et al.^8^ who displayed ultrasonic propagation through dental tissue. Transmission line theory was employed to model the longitudinal waves produced by the ultrasonic transducer propagating through an infinite medium. Due to the one-dimensional nature of the PSpice program, the data obtained resembles those of ultrasonic A-scan signatures collected at specific locations along the propagation pathway. The simulation enables the evaluation of the propagated signal from both the cervical and site-focused stimulation treatments for sufficient intensity to give rise to an action potential.

## Methods

**Figure 1:**
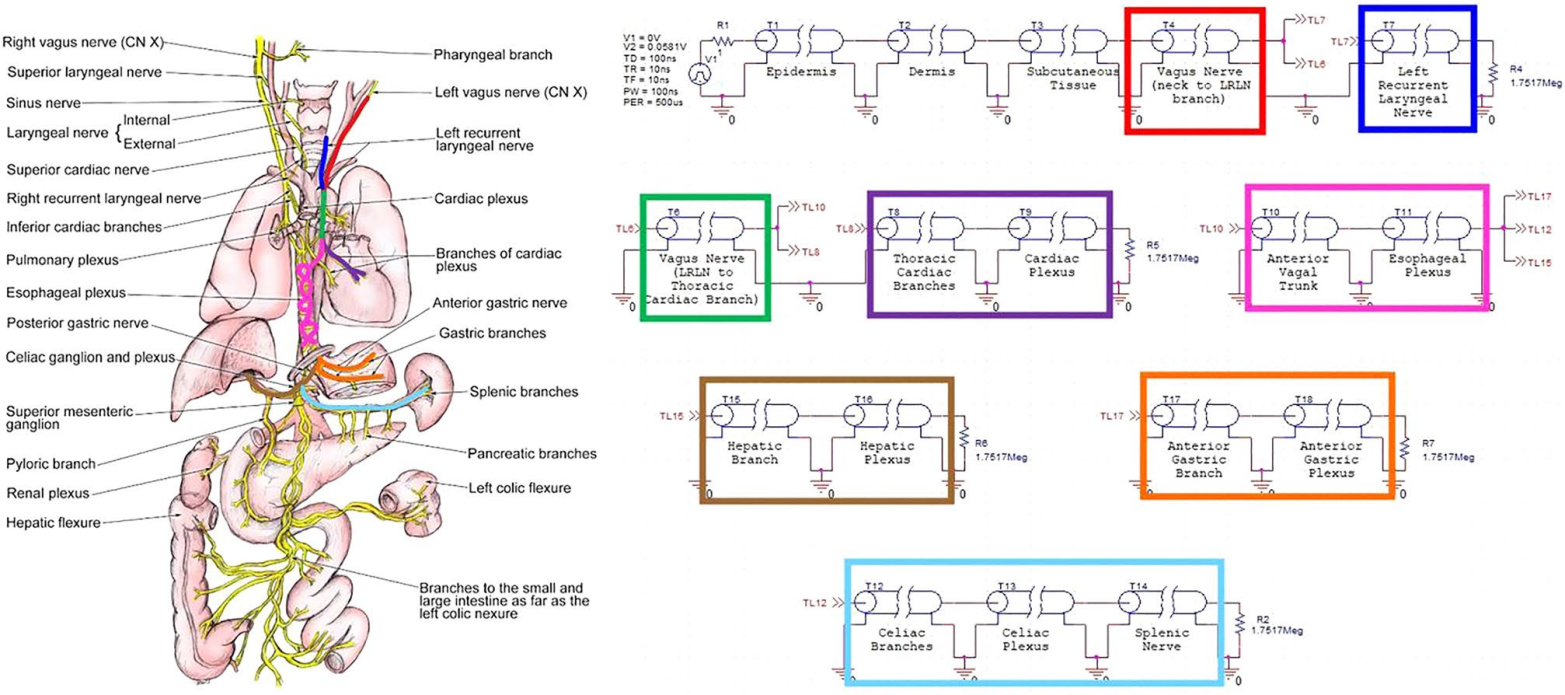
Vagus nerve map with colored lines illustrating the path of the PSpice implementation of cervical ultrasonic neurostimulation. Left image of vagus nerve branching adapted and reproduced with permission from Medscape Drugs & Diseases (https://emedicine.medscape.com/), Vagus Nerve Anatomy, 2017, available at: https://emedicine.medscape.com/article/1875813-overview^10^.

**Figure 2:**
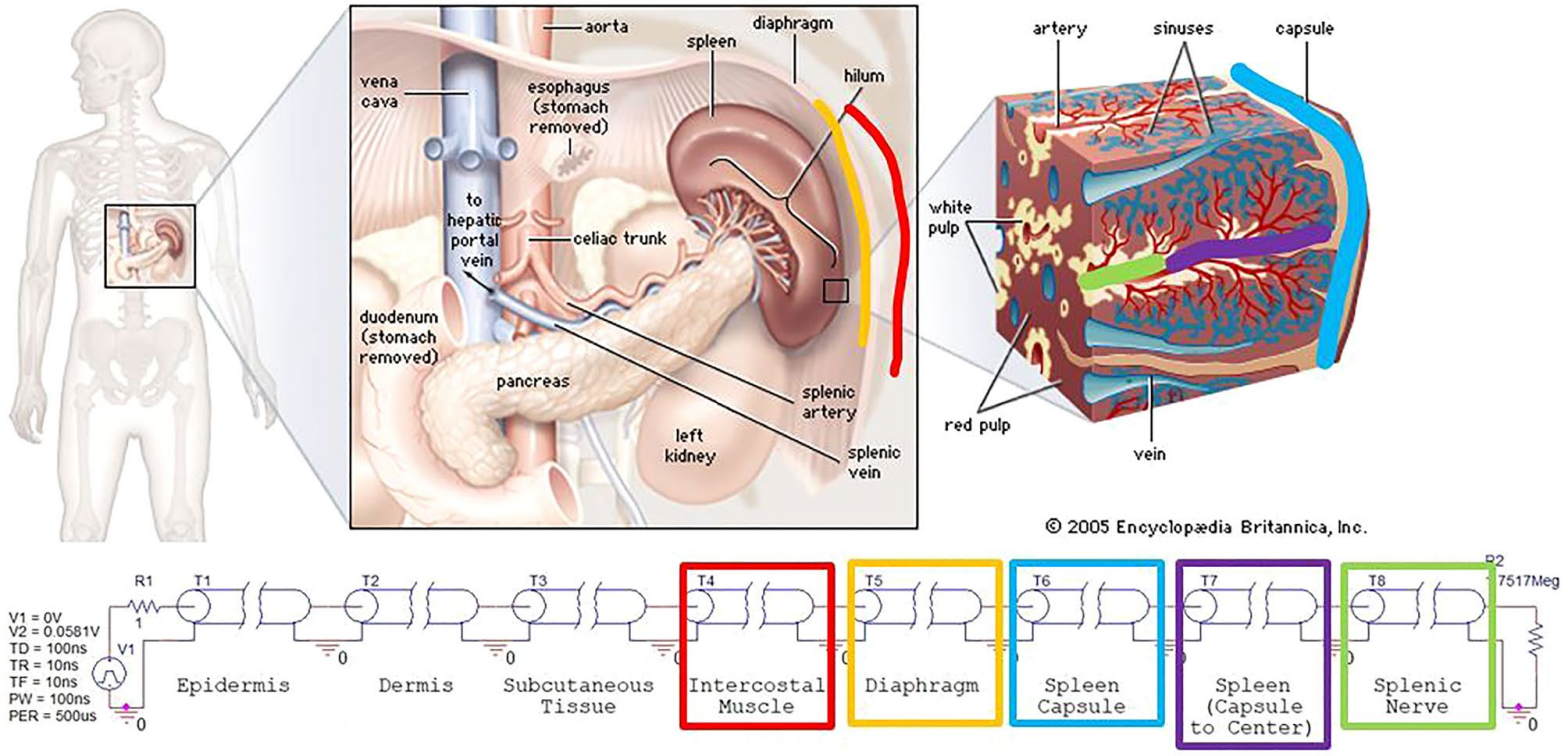
PSpice implementation of site-focused splenic ultrasonic neurostimulation. Top image of spleen anatomy by courtesy of Encyclopædia Britannica, Inc., copyright 2009; used with permission^11^.

In order to simulate ultrasonic neurostimulation, acoustic properties of the ultrasonic waves were converted to electrical characteristics in an electro-acoustical analogy using PSpice (Orcad™; Hillsboro OR). The voltage values obtained in the simulation equate to the pressure responses at each tissue interface as discussed later. The neurostimulation to reduce inflammation goes through the vagus nerve as described above. Two scenarios were generated, a cervical and a site-focused model. The cervical model, seen in Fig. 1, introduced the ultrasonic waves at the neck region of the vagus nerve, propagating along the vagus nerve to the spleen, while bifurcating through the efferent neural pathways of the vagus nerve, such as the heart, liver, and stomach. The site-focused model, seen in Fig. 2, involved the stimulation site being above the spleen, at the skin level of the abdomen. Therefore, there were no efferent pathways for the signal to bifurcate into.

Each transmission line served as a different layer of tissue. Each tissue type in the body had its own acoustic properties: acoustic impedance, speed of sound, and density. As an acoustic wave propagates through the body, these properties change and affect how the wave will be attenuated. Given that the model served to represent a therapeutic application of ultrasound, a transducer was not included in the model. A pulsed voltage supply with a period of 500 μs was used as the incident source and the signal was measured at the terminals of each branch^1^. Transmission lines were used to model nerve fibers as these were sufficient to store and transmit a signal from the receiving to the terminating end^9^. The resistor placed at the end of each branch matched the acoustic impedance of the transmission line terminal located right before. This accomplished impedance matching to reduce unwanted reflections.

The transmission lines are the electrical elements labeled T* in Figs. 1 and 2, where the “*” represents the number associated with each transmission line terminal. These elements have two pins on each side, as shown in Supplementary Fig. S2. The left pins labeled A + and A − are for the input signal. The right pins labeled B + and B − are for the output signal. The two negative pins are connected to ground. Therefore, the positive pins were the ones needed to measure the voltage values in the propagation pathway with respect to ground. The B + pin was used to measure the voltage at the end of specific tissue nerve segments in the model with the notation *v*(T*:B +).

The model allowed for the branching of the vagus nerve in the cervical model as seen in Fig. 1. The initial pulse was sent through the layers of the skin: epidermis, dermis, and subcutaneous tissue. Following the skin, the wave propagated through the vagus nerve before reaching the first branch at the left recurrent laryngeal nerve (LRLN), highlighted in red. The LRLN was highlighted in royal blue. The model continued through the vagus nerve until it reached another branch between the thoracic cardiac branch and the anterior vagal trunk, highlighted in green. The thoracic cardiac branch terminated at the cardiac plexus, highlighted in purple. The anterior vagal trunk connected to the esophageal plexus, highlighted in pink, where another branch occurred for the acoustic wave to split. This intersection split three ways, one of which went to the hepatic branch before terminating at the hepatic plexus, highlighted in brown. Another branch led to the anterior gastric branch before terminating at the anterior gastric plexus, highlighted in orange. Lastly, the third branch led to the celiac branch, the celiac plexus, and the splenic nerve, highlighted in light blue. All connections between elements were grounded.

The site-focused model, shown in Fig. 2, simulated the stimulation of the same splenic nerve. However, the incident wave originated directly above the spleen through the abdomen, rather than originating from the neck. For this reason, the model went linearly, simulating the wave passing through the three layers of the skin, the intercostal muscle (red), the diaphragm (orange), the spleen capsule (light blue), the spleen (purple), and ultimately the splenic nerve (green).

The PSpice models for both cases used a transient, time domain analysis. The cervical simulation ran for 800 μs while the site-focused simulation ran for 100 μs. These value were determined based on the time needed to see one pulse at each transmission line terminal. They were made to be slightly larger than the longest propagation time observed. A pulsed voltage source with a period of 500 μs was used to mimic that of the ultrasonic transducer. The voltage supplied was based on the experiment conducted by Cotero et al.^1^. The 1.1 MHz Sonic Concepts H106 transducer used in the experiment was calibrated with an ONDA HNA-0400 needle hydrophone. The hydrophone had a nominal sensitivity of 70 nV/Pa^12^. The optimal mechanical pressures reported were 0.25 and 0.83 MPa^1^. The conversion from pressure to voltage was calculated using Eq. (1)^13^:

1 $$ p\left( t \right) = \frac{V\left( t \right)}{M} $$

where $p\left( t \right)$ is the acoustic pressure waveform, $V\left( t \right)$ is the hydrophone voltage, and $M$ is the hydrophone sensitivity. Given the pressure and the sensitivity above, the voltages were calculated to be 17.5 and 58.1 mV, respectively. The acoustic impedance for each tissue section was calculated using Eq. (2)^14^:

2 $$ Z = \rho c $$

where $Z$ is acoustic impedance, $\rho$. is density of the tissue, and $c$ is the speed of sound within that tissue. The time delay was calculated using the known relationship between distance and velocity using:

3 $$ t = \frac{d}{c} $$

where $t$ is the time delay and $d$ is the thickness/length of the tissue. The tissue characteristics were obtained from established acoustic properties. The length of some nerve portions were approximated based on human anatomy. The tissue properties are summarized in Supplementary Tables S1 and S2^15–23^.

## Results

The simulation resulted in a set of data representing the longitudinal waves from the ultrasonic stimulation. The PSpice models shown in Figs. 1 and 2 generated one-dimensional A-scan waveforms along the propagation pathway at the interface between any two adjacent tissue layers and elsewhere. However, the only signals of interest were at the final transmission line terminal of each branch where it no longer bifurcates. Due to the nature of the analysis, the voltage signals needed to represent the propagation through the tissue layers were on the output side of the transmission line labeled B + . The measured voltage signals are annotated as *v*(T*:B +), where “*” represents the transmission line number.

**Figure 3:**
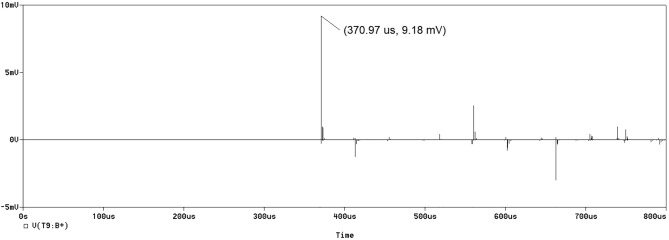
A-scan signal produced by the 0.25 MPa PSpice simulation. This measured voltage from the cervical stimulation was obtained at *v*(T9:B +) for the cardiac plexus.

For the cervical model, shown in Fig. 1, several voltages were measured, such as *v*(T7:B +) for the left recurrent laryngeal nerve (LRLN), *v*(T9:B +) for the cardiac plexus, *v*(T16:B +) for the hepatic plexus, *v*(T18:B +) for the gastric plexus, and *v*(T14:B +) for the splenic nerve. However, for the site-focused model, shown in Fig. 2, only the voltage, *v*(T8:B +), was measured. This was due to the nature of the site-focused model. Since the model was only targeting the splenic nerve, there were no other branches at which to measure the terminal voltage. The measured voltage values represent the amplitude of the A-scan signatures of the ultrasonic wave as it propagates and interacts with the different interfaces of tissue layers. For each waveform generated, the first peak was the primary pulse released by the voltage source (transducer). The subsequent peaks were due to reflections, as discussed later. A sample output of the transmission line simulation representing the A-scan for the cardiac plexus in the cervical model is shown in Fig. 3. The remaining A-scans associated with the other nerve segments can be found in Supplementary Fig. S3 and S4. The locations of the organs were known based on anatomical dimensions incorporated into the PSpice model. The PSpice models were evaluated for acoustic pressures of 0.25 and 0.83 MPa.

### Acoustic pressure 0.25 MPa

**Figure 4:**
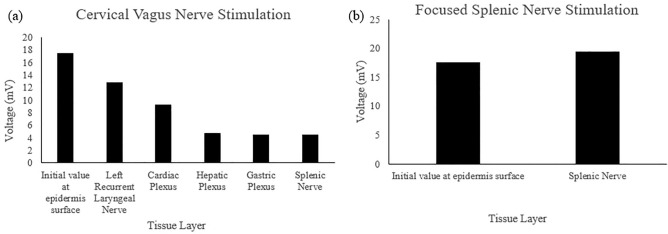
Voltage values obtained from the 0.25 MPa simulation A-scans for each tissue layer for both the (**a**) cervical vagus nerve and (**b**) focused splenic nerve simulations.

For the 0.25 MPa acoustic pressure, the signal arriving at the LRLN propagated for 708.52 μs and delivered a voltage of 12.84 mV. The signal arriving at the cardiac plexus propagated for 370.97 μs and delivered a voltage of 9.18 mV. The signal to the hepatic plexus propagated for 523.09 μs and delivered a voltage of 4.64 mV. The signal to the gastric plexus propagated for 492.40 μs and delivered a voltage of 4.45 mV. The signal to the splenic nerve through the cervical PSpice model, had a propagation time of 504.67 μs and a voltage of 4.43 mV. The site-focused PSpice model showed the propagation time to the splenic nerve at 57.55 μs and a delivered voltage of 19.39 mV. These values are depicted in Fig. 4 and summarized in Table 1 along with the values used for the initial site. The individual A-scans can be found in Supplementary Fig. S3.

**Table 1:** Summarized results of 0.25 MPa cervical and site-focused simulations.

Tissue	Transmission line terminal	Propagation time (μs)	Voltage (mV)	Mechanical pressure (MPa)	Intensity (W/cm^2^)	Attenuation (dB)
**Cervical vagus nerve stimulation**						
Initial value at epidermis surface	*v*(T1:A +)	0	17.5	0.25	1.86	0
Left Recurrent Laryngeal Nerve	*v*(T7:B +)	708.52	12.84	0.18	0.96	− 2.69
Cardiac Plexus	*v*(T9:B +)	370.97	9.18	0.13	0.49	− 5.60
Hepatic Plexus	*v*(T16:B +)	523.09	4.64	0.07	0.13	− 11.54
Gastric Plexus	*v*(T18:B +)	492.40	4.45	0.06	0.12	− 11.88
Splenic Nerve	*v*(T14:B +)	504.67	4.43	0.06	0.11	− 11.93
**Focused splenic nerve stimulation**						
Initial value at epidermis surface	*v*(T1:A +)	0	17.5	0.25	1.86	0
Splenic Nerve	*v*(T8:B +)	57.55	19.39	0.28	2.19	0.89

### Acoustic pressure 0.83 MPa

**Figure 5:**
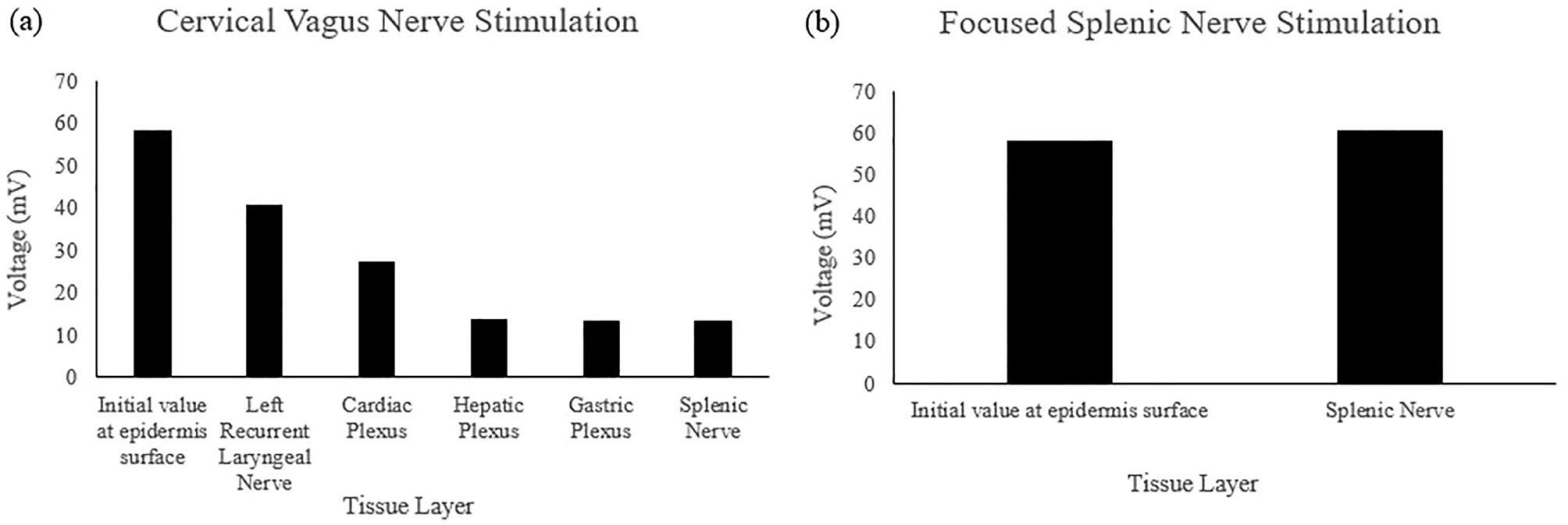
Voltage values obtained from the 0.83 MPa simulation A-scans for each tissue layer for both the (**a**) cervical vagus nerve and (**b**) focused splenic nerve simulations.

For the 0.83 MPa acoustic pressure, the signal arriving at the LRLN propagated for 708.51 μs and delivered a voltage of 40.37 mV. The signal arriving at the cardiac plexus propagated for 370.96 μs and delivered a voltage of 26.96 mV. The signal to the hepatic plexus propagated for 523.08 μs and delivered a voltage of 13.48 mV. The signal to the gastric plexus propagated for 492.45 μs and delivered a voltage of 13.18 mV. The signal to the splenic nerve through the cervical PSpice model, had a propagation time of 504.72 μs and a voltage of 13.18 mV. The site-focused PSpice model showed the propagation time to the splenic nerve at 57.53 μs and a delivered voltage of 60.66 mV. These values are depicted in Fig. 5 and summarized in Table 2 along with the values used for the initial site. The individual A-scans can be found in Supplementary Fig. S4.

**Table 2:** Summarized results of 0.83 MPa cervical and site-focused simulations.

Tissue	Transmission line terminal	Propagation time (μs)	Voltage (mV)	Mechanical pressure (MPa)	Intensity (W/cm^2^)	Attenuation (dB)
**Cervical vagus nerve stimulation**						
Initial value at epidermis surface	*v*(T1:A +)	0	58.1	0.83	20.53	0
Left Recurrent Laryngeal Nerve	*v*(T7:B +)	708.51	40.37	0.58	9.49	− 3.16
Cardiac Plexus	*v*(T9:B +)	370.96	26.96	0.39	4.23	− 6.67
Hepatic Plexus	*v*(T16:B +)	523.08	13.48	0.19	1.06	− 12.69
Gastric Plexus	*v*(T18:B +)	492.45	13.18	0.19	1.01	− 12.89
Splenic Nerve	*v*(T14:B +)	504.72	13.18	0.19	1.01	− 12.89
**Focused splenic nerve stimulation**						
Initial value at epidermis surface	*v*(T1:A +)	0	58.1	0.83	20.53	0
Splenic Nerve	*v*(T8:B +)	57.53	60.66	0.87	21.43	0.37

### Ultrasonic intensity

The corresponding intensities were calculated for each terminal branch. The intensity was calculated using Eq. (4)^6^:

4 $$ I_{SPTA} = \frac{{p^{2} }}{2\rho c} $$

where $I_{SPTA}$ is the spatial peak temporal average intensity, $p$ is pressure, $\rho$ is density, and $c$ is the speed of sound in tissue. The mechanical pressure was calculated using Eq. (1) with the same hydrophone sensitivity. This was applied to the calculation for voltage to pressure, at the tissue level, enabling the results to be relative to the initial values. Additionally, it represents the intensity as if the transducer was placed directly on the tissue. For the 0.25 MPa simulation, the intensities for the cervical model terminals, *v*(T7:B +), *v*(T9:B +), *v*(T16:B +), *v*(T18:B +), and *v*(T14:B +) were 0.96, 0.49, 0.13, 0.12, and 0.11 W/cm^2^, respectively (Table 1). The intensity for the site-focused model at the splenic nerve, *v*(T8:B +), was 2.19 W/cm^2^ (Table 1). For the 0.83 MPa simulation, the intensities for the cervical model terminals, *v*(T7:B +), *v*(T9:B +), *v*(T16:B +), *v*(T18:B +), and *v*(T14:B +) were 9.49, 4.23, 1.06, 1.01, and 1.01 W/cm^2^, respectively (Table 2). The intensity for the site-focused model splenic nerve, *v*(T8:B +), was 21.43 W/cm^2^ (Table 2).

### Attenuation

The attenuation of the acoustic wave was calculated at each transmission line terminal relative to the initial value and computed using Eq. (5):

5 $$ Attenuation \left( {dB} \right) = 20{\text{log}}\left( {\frac{{v\left( {T^{*} :{\text{B}} + } \right)}}{{v\left( {T1:A + } \right)}}} \right) $$

where again *v*(T*:B +) is the voltage obtained at each transmission line with “*” being the transmission line number and where *v*(T1:A +) is the incident voltage of the ultrasound wave discussed earlier (17.5 and 58.1 mV for the 0.25 and 0.83 MPa simulations, respectively). For the 0.25 MPa case, the attenuations for the cervical model terminals, *v*(T7:B +), *v*(T9:B +), *v*(T16:B +), *v*(T18:B +), and *v*(T14:B +) were − 2.69, − 5.60, − 11.54, − 11.88, and − 11.93 dB, respectively (Table 1). The attenuation for the site-focused model at the splenic nerve, *v*(T8:B +), was 0.89 dB (Table 1). For the 0.83 MPa simulation, the attenuations for the cervical model terminals, *v*(T7:B +), *v*(T9:B +), *v*(T16:B +), *v*(T18:B +), and *v*(T14:B +) were − 3.16, − 6.67, − 12.69, − 12.89, and − 12.89 dB, respectively (Table 2). The attenuation for the site-focused model at the splenic nerve, *v*(T8:B +), was 0.37 dB (Table 2).

## Discussion

The ultrasound wave underwent a loss in the cervical model as indicated by the negative attenuation while the site-focused model showed a gain as reflected by the positive attenuation. This occurred for both the 0.25 and 0.83 MPa simulations. The signal leaving the LRLN had the largest amplitude compared to the other terminals in the cervical model. This was a result of the branch anatomy. The farther along the vagus nerve the signal propagated, the larger the number of bifurcations the signal was divided into, decreasing the terminal signal measured and thereby increasing the loss. For the LRLN, the 0.25 MPa simulation signal was attenuated from 17.5 mV to 12.84 mV and the 0.83 MPa simulation signal was attenuated from 58.1 mV to 40.37 mV, yielding a loss of 2.69 and 3.16 dB, respectively. The smaller peaks seen after the initial peak were due to reflections. The transmission lines were designed so the signal propagating through them would reflect back to the origin. This resulted in constructive and destructive interference with the initial wave pulse. While this seems unfavorable, it aids in the modeling of acoustic waves in the body as indicated by the reflection of these waves off of tissue boundaries, interfering with the incident waves. The propagation time was dependent on the length of nerve segments. Therefore, sections with larger lengths had higher propagation times, regardless of the terminal voltage. The LRLN in both pressure models had approximately the same propagation time of 708 μs because it was not dependent on the incident amplitude.

The cardiac branch similarly saw attenuation of the signal from the initial voltage delivered to the final voltage seen at the cardiac plexus. For the 0.25 MPa simulation, the signal was attenuated from 17.5 mV to 9.18 mV, yielding a loss of 5.60 dB. For the 0.83 MPa simulation, the signal was attenuated from 58.1 mV to 26.96 mV, yielding a loss of 6.69 dB. Likewise, these waveforms illustrated the wave reflections associated with the transmission lines. The propagation time was approximately the same for both pressure simulations at 371 μs. This was shorter compared to the LRLN branch due to the lengths of branches the signal was transmitted through. The attenuation was larger as the signal had to propagate further along the pathway. The hepatic branch saw the same effects with the 0.25 MPa signal being attenuated to 4.64 mV and the 0.83 MPa signal being attenuated to 13.48 mV, yielding losses of 11.54 and 12.69 dB, respectively. The shared propagation time was approximately 523 μs. The gastric branch had the signal attenuated to 4.45 and 13.18 mV in the 0.25 and 0.83 MPa simulations, respectively, with an approximate propagation time of 492 μs. The gastric signals had losses of 11.88 and 12.89 dB, respectively. Lastly, the splenic nerve saw the signal attenuated to 4.43 and 13.18 mV in the 0.25 and 0.83 MPa simulations, respectively, with a 504 μs propagation time. The splenic nerve losses were 11.93 and 12.89 dB, respectively.

The site-focused models registered an increase in the terminal signal at the splenic nerve compared to the initial signal delivered for both pressure simulations. The signal increased from 17.5 to 19.39 mV for the 0.25 MPa simulation, yielding a gain of 0.89 dB. For the 0.83 MPa simulation, the signal increased from 58.1 to 60.66 mV, yielding a gain of 0.37 dB. The increase in voltage was due to the constructive interference resulting from the waves reflecting back in the transmission lines. The propagation time for both pressure models was approximately 57 μs. Given that the site-focused model contains fewer and shorter tissue segments, the propagation time was far shorter than that of any terminal in the cervical model. To evaluate efficacy, the voltages for each terminal were converted to intensities.

As stated earlier, a minimum intensity of 0.1 W/cm^2^ was required to initiate an action potential, according to the NICE model. After analyzing the signals received in both pressure simulations, an action potential would generate in each of the branches in the cervical and site-focused models for each pressure profile. These action potentials may be irritating/detrimental to the function of some of the tissue/organs. The branch innervating the laryngeal muscles (LRLN) had an intensity of 0.96 and 9.49 W/cm^2^ for the 0.25 and 0.83 MPa simulations, respectively. Exciting the larynx induces hoarseness and a tingling sensation in the throat^24^. The branch innervating the heart had an intensity of 0.49 and 4.23 W/cm^2^ for the 0.25 and 0.83 MPa simulations, respectively. This excitation decreases heart rate^1^. The branch innervating the liver had an intensity of 0.13 and 1.06 W/cm^2^ for the 0.25 and 0.83 MPa simulations, respectively. The liver being excited increases blood glucose levels and inhibits insulin secretion^25^. The excitation of the gastric plexus had an intensity of 0.12 and 1.01 W/cm^2^ for the 0.25 and 0.83 MPa simulations, respectively and leads to accelerated gastric emptying through relaxation of the pyloric sphincter^26^. Ultimately, the splenic nerve, when stimulated, reduces the circulation of certain inflammatory cytokines (TNF-α) through the mechanism described above. The cervical model generated an intensity of 0.11 and 1.01 W/cm^2^ for the 0.25 and 0.83 MPa simulations, respectively. For the site-focused model, the intensity was 2.19 and 21.43 W/cm^2^ for the 0.25 and 0.83 MPa simulations, respectively. In the site-focused model, the acoustic wave only traveled in the focused region of the spleen and the splenic nerve, rather than the entire vagus nerve. Therefore, the site-focused model reduces these efferent effects to the other biological systems associated with cervical stimulation.

When discussing ultrasound as a therapeutic tool, it is imperative that the intensity of the wave must be within set parameters to ensure patient safety. The FDA guidelines for therapeutic ultrasound dictate that the intensity must not exceed 3 W/cm^2^ to ensure harmful heating does not occur in tissue^27^. Therefore, according to the 0.83 MPa simulation (Table 2), the signals delivered to the LRLN and heart in the cervical model, and the splenic nerve in the site-focused model would violate this safety standard and cause more harm to the LRLN and the heart before the wave continues to propagate through the vagus nerve. The study in which the acoustic pressures were obtained dictated the threshold for safety from thermal burns was 35 W/cm^2^^1^. Given this value, the 0.83 MPa treatment would not exceed the threshold. However, 35 W/cm^2^ does not align with the FDA standards and thus the 0.83 MPa treatment was not viable. Conversely, in the 0.25 MPa simulation, all of the intensity values for both the cervical and site-focused models did not exceed the maximal threshold value of 3 W/cm^2^, meeting the standard.

While the safety surrounding the 0.83 MPa was not valid, the effectiveness in exciting an action potential can be supported with the physical experiment conducted by Cotero et al.^1^. They indicated that the heart rate decreased up to 20% after cervical VNS while the site-focused stimulation treatment, at the spleen, did not see a change in heart rate (Supplementary Fig. S5). This supports the hypothesis that the cervical model will lead to greater undesired efferent effects compared to the site-focused model. Furthermore, they demonstrated that in inflamed subjects, the focused treatment with 0.25 MPa pressure increased splenic norepinephrine and acetylcholine while decreasing splenic TNF-α and whole blood TNF-α (Supplementary Fig. S6). A similar result was seen with the 0.83 MPa group, however this did not meet the FDA standard, according to the 0.83 MPa PSpice simulation. These groups were compared to other acoustic pressures as well as naïve groups and sham groups. The naïve group did not have any conditions introduced while the sham group had an induced state of inflammation but was not given ultrasonic neurostimulation treatment. In Supplementary Fig. S6, the whole blood TNF-α concentration for the 1.72 MPa ultrasonic treatment saw a drastic increase compared to the other treatment pressures. This was likely due to the intensity for this pressure being largely over the FDA standard of 3 W/cm^2^, leading to thermal burns in the tissue. Instead of reducing inflammation by exciting the splenic nerve, the treatment resulted in tissue damage that necessitated innate inflammation for healing. Ultimately, a safe acoustic pressure was required to reduce inflammation in patients with amplified states.

Autoimmune diseases increase a person's state of inflammation even if their body is healthy. These diseases target the body itself initiating an inflammatory response. As the body keeps fighting itself, the inflammation remains. A treatment such as splenic site-focused pulsed ultrasonic neurostimulation can help alleviate the pain associated with the chronic inflammatory state by reducing the TNF-α production. By decreasing TNF-α production through the excitation of the cholinergic anti-inflammatory pathway, it will not be able to provoke additional inflammatory molecules by binding to circulating macrophages.

Ultrasonic stimulation of the vagus nerve is a complex process that involves many biological systems. The use of a one-dimensional electrical modeling program simplifies the mechanism for analysis. The PSpice simulations were successful in illustrating the anticipated attenuation of the acoustic waves while preserving the required intensity to initiate an action potential. Furthermore, it supported that the site-focused treatment prevents efferent pathways of the vagus nerve from being stimulated unnecessarily. Overall, the use of this electrical modeling software to simulate an ultrasonic treatment of biological tissue allowed for the evaluation of three main factors: design, safety, and versatility. The program allowed for the system to be modelled to include certain visceral areas and exclude others. It enabled for the ultimate intensities to be calculated and ensure they met FDA standards. It supported the versatility in that more than one treatment option can be designed in the space. A model such as this can be applied to future work to fine tune ultrasonic neurostimulation systems and has the potential to contribute to the discovery of the functioning mechanism that yields action potentials from incident ultrasonic waves.

## Supplementary Information

Supplementary Information.

## Data Availability

All data is available on reasonable request, directed to the corresponding author, Sleiman R. Ghorayeb (Sleiman.R.Ghorayeb@hofstra.edu).
