# Supplementary material for: PSpice modeling of cervical and site-focused vagus nerve ultrasonic stimulation for reduced tumor necrosis factor-α production
Source: Sci Rep. 2022 Dec 12;12:21470. doi: 10.1038/s41598-022-25944-2 (PMC9744857; doi:10.1038/s41598-022-25944-2)
Supplement: Supplementary file 1 — Supplementary Information. [file 41598_2022_25944_MOESM1_ESM.pdf]

PSpice Modeling of Cervical and Site-Focused Vagus Nerve Ultrasonic Stimulation for Reduced Tumor Necrosis Factor-α Production

Sleiman R. Ghorayeb and Bryan Hirsch

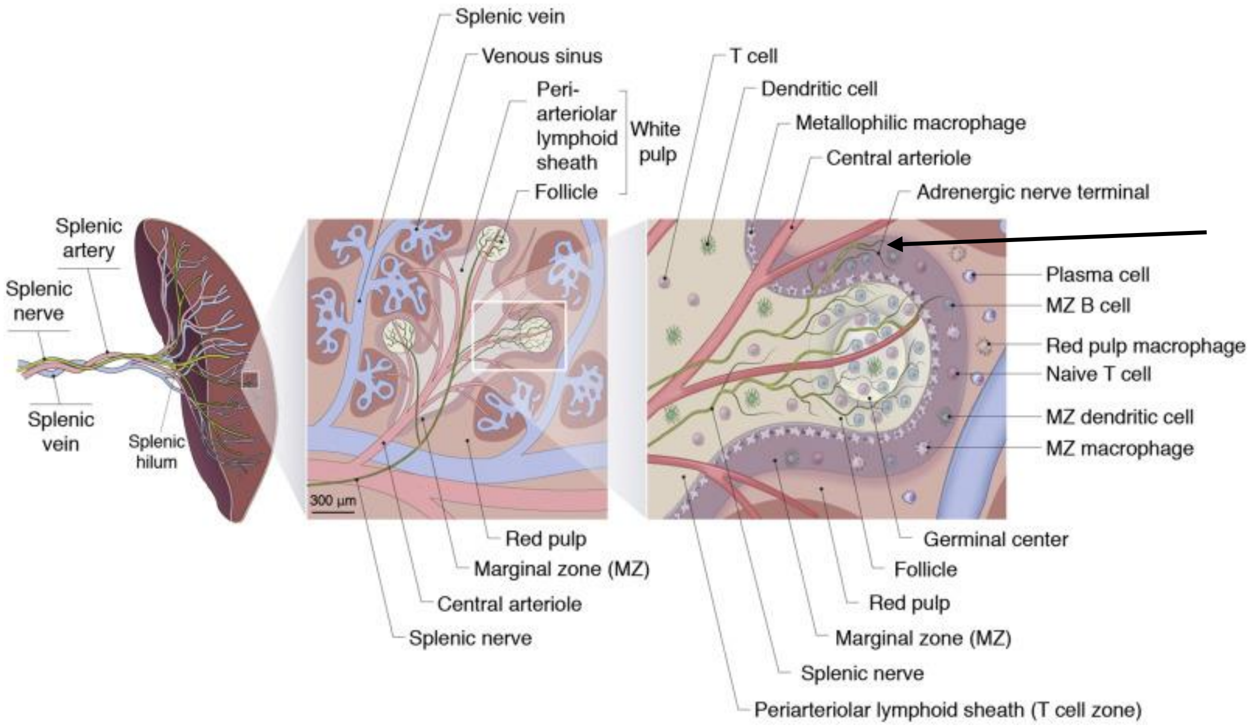

Supplementary Figure S1. Spleen anatomy illustrating adrenergic nerve terminal location. The arrow is pointing to one of these specific locations within the marginal zone. Adapted and reproduced with permission from Elsevier. [28]

[28] Noble, B. T., Brennan, F. H. & Popovich, P. G. The spleen as a neuroimmune interface after spinal cord injury. *J.Neuroimmunol.* **321**, 1–11 (2018).

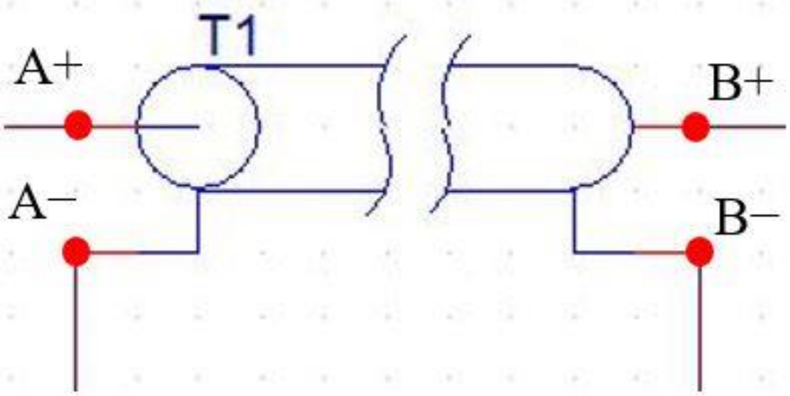

Supplementary Figure S2. PSpice transmission line element with labeled pins A+, A−, B+, and B−

Material Properties of Tissue in Cervical Vagus Nerve Stimulation

Tissue	Speed of Sound (m/s)	Thickness/ Length (μm)	Density (kg/m^3)	Time Delay (μs)	Acoustic Impedance (MRayls)
Epidermis (Lateral Neck)	1645	32.89	1020	0.02	1.68
Dermis (Lateral Neck)	1595	1440.71	1020	0.90	1.63
Subcutaneous Tissue (Lateral Neck)	1440.2	29000	900	20.14	1.30
Vagus Nerve (neck to LRLN branch)	1629.5	120000	1075	73.64	1.75
Left recurrent laryngeal nerve	1629.5	1000000	1075	613.69	1.75
Vagus Nerve (LRLN branch to thoracic cardiac branch)	1629.5	300000	1075	184.11	1.75
Thoracic Cardiac Branches (TCB)	1629.5	100000	1075	61.37	1.75
Cardiac Plexus	1629.5	50000	1075	30.68	1.75
Anterior Vagal Trunk	1629.5	187900	1075	115.31	1.75
Esophageal Plexus	1629.5	50000	1075	30.68	1.75
Hepatic Branch	1629.5	120000	1075	73.64	1.75
Hepatic Plexus	1629.5	40000	1075	24.55	1.75
Anterior Gastric Branch	1629.5	60000	1075	36.82	1.75
Anterior Gastric Plexus	1629.5	50000	1075	30.68	1.75
Celiac Branch	1629.5	60000	1075	36.82	1.75
Celiac Plexus	1629.5	20000	1075	12.27	1.75
Splenic Nerve	1629.5	50000	1075	30.68	1.75

Supplementary Table S1. Material Properties of Tissue in Cervical Vagus Nerve Stimulation

Material Properties of Tissue in Splenic Site-Focused Nerve Stimulation

Tissue	Speed of Sound (m/s)	Thickness/ Length (μm)	Density (kg/m^3)	Time Delay (μs)	Acoustic Impedance (MRayls)
Epidermis	1645	32.89	1020	0.02	1.68
Dermis	1595	1440.71	1020	0.90	1.63
Subcutaneous Tissue	1440.2	29000	900	20.14	1.30
Intercostal Muscle	1588.4	3300	1090	2.08	1.73
Diaphragm	1588.4	1500	1090	0.94	1.73
Spleen Capsule	1567.6	125.71	1089	0.08	1.71
Spleen (Capsule to Center)	1567.6	22000	1089	14.03	1.71
Splenic Nerve	1629.5	2000	1075	1.23	1.75

Supplementary Table S2. Material Properties of Tissue in Splenic Site-focused Ultrasonic Nerve Stimulation

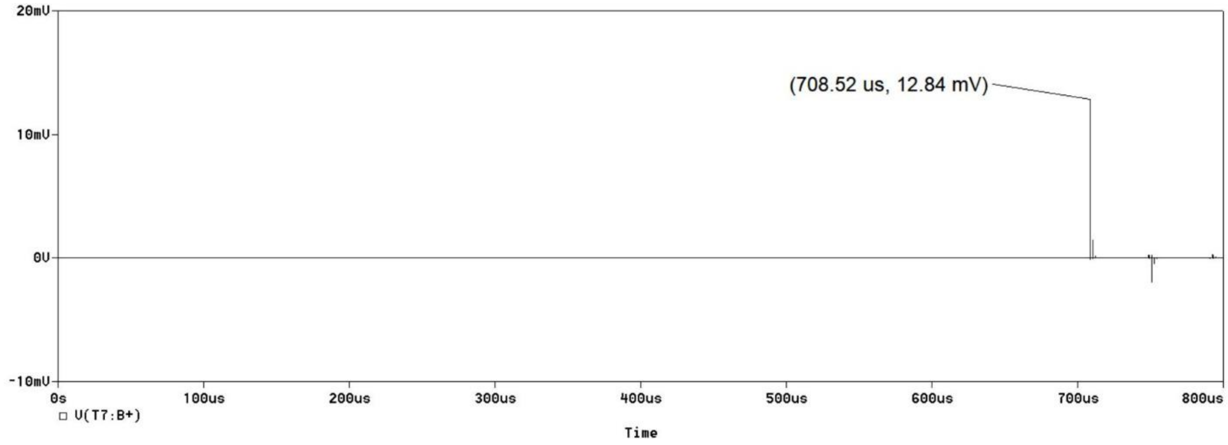

20mV
10mV
0V
-10mV
(708.52 us, 12.84 mV)
0s
100us
200us
300us
400us
500us
600us
700us
800us
V(T7:B+)
Time

(a)

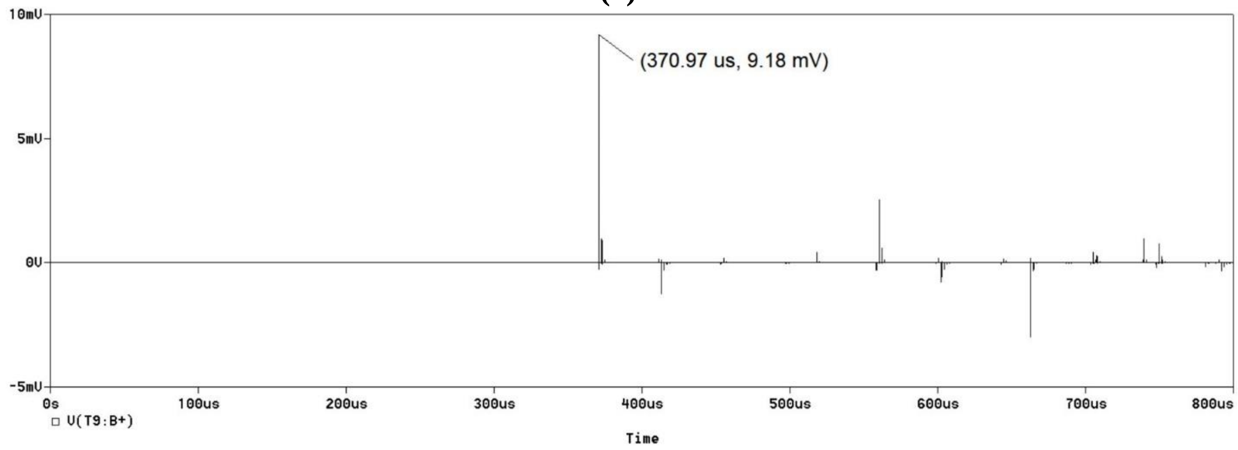

10mV
5mV
0V
-5mV
(370.97 us, 9.18 mV)
0s
100us
200us
300us
400us
500us
600us
700us
800us
V(T9:B+)
Time

(b)

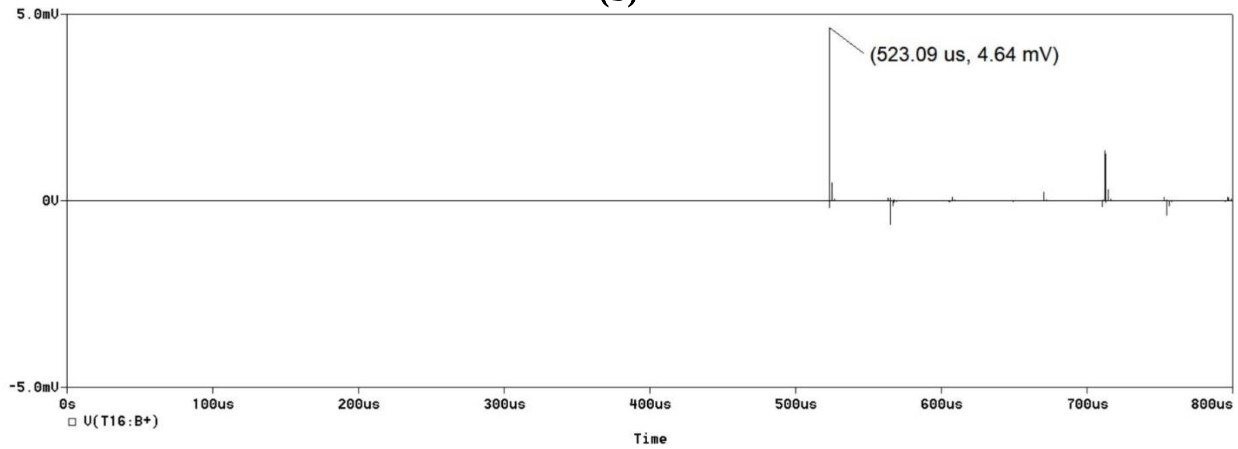

5.0mV
0V
-5.0mV
(523.09 us, 4.64 mV)
0s
100us
200us
300us
400us
500us
600us
700us
800us
V(T16:B+)
Time

(c)

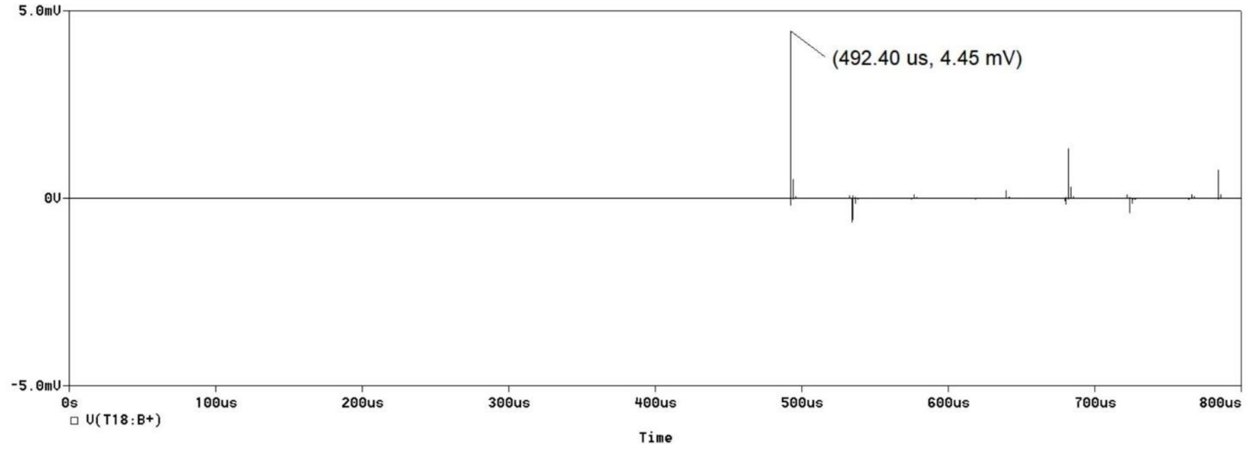

(d)

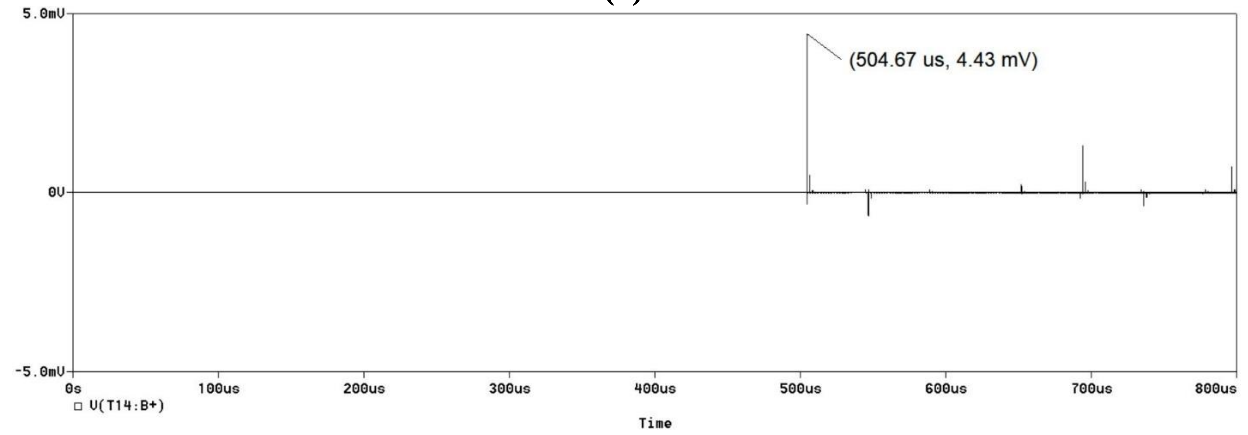

(e)

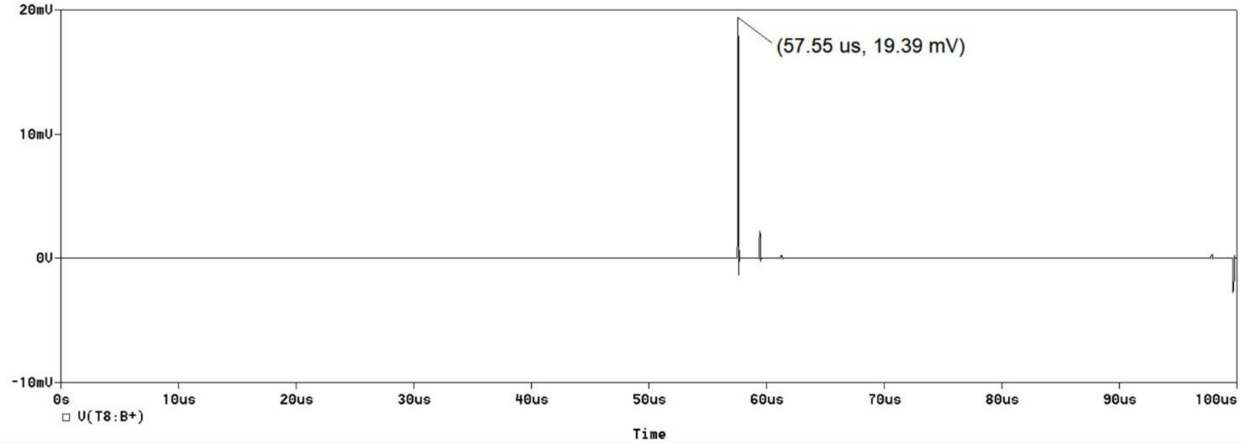

(f)

Supplementary Figure S3. A-scan signals produced in 0.25 MPa PSpice simulation. The measured voltages from the cervical simulation are seen in (a) *v*(T7:B+) for the left recurrent laryngeal nerve, (b) *v*(T9:B+) for the cardiac plexus, (c) *v*(T16:B+) for the hepatic plexus, (d) *v*(T18:B+) for the gastric plexus, and (e) *v*(T14:B+) for the splenic nerve. The measured voltage from the site-focused simulation is seen in (f) *v*(T8:B+) for the splenic nerve

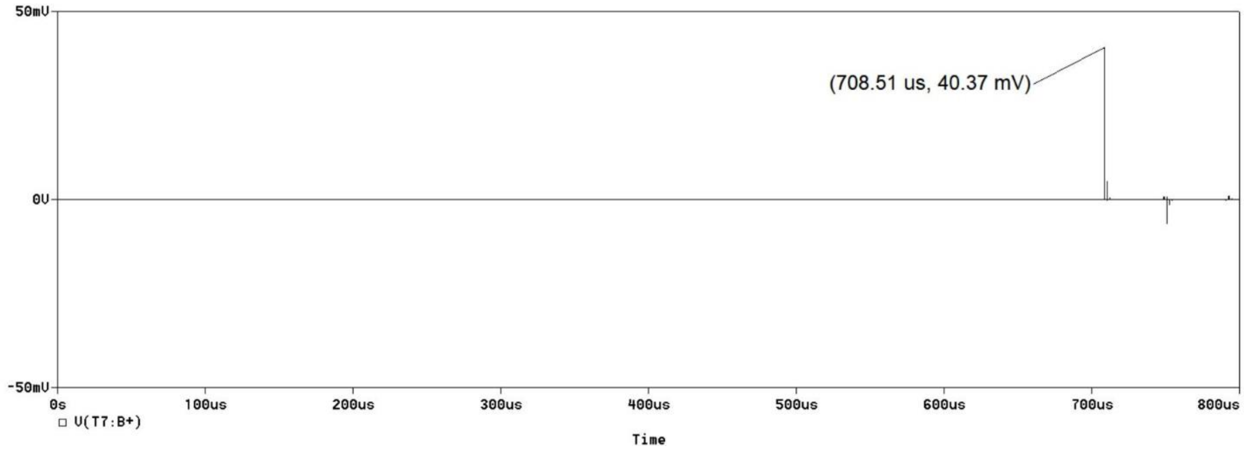

50mV
0V
-50mV
(708.51 us, 40.37 mV)
0s
100us
200us
300us
400us
500us
600us
700us
800us
V(T7:B+)
Time

(a)

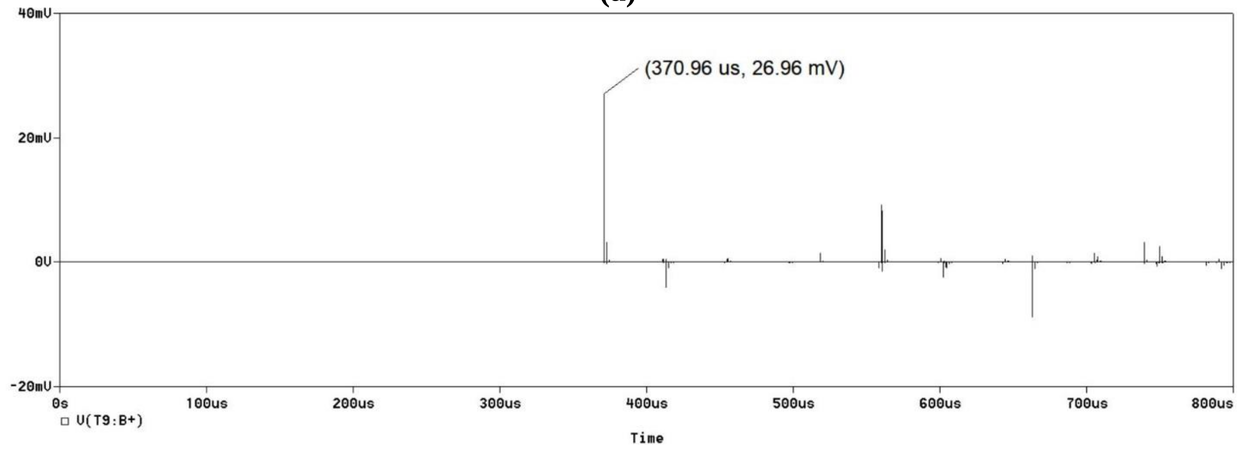

40mV
20mV
0V
-20mV
(370.96 us, 26.96 mV)
0s
100us
200us
300us
400us
500us
600us
700us
800us
V(T9:B+)
Time

(b)

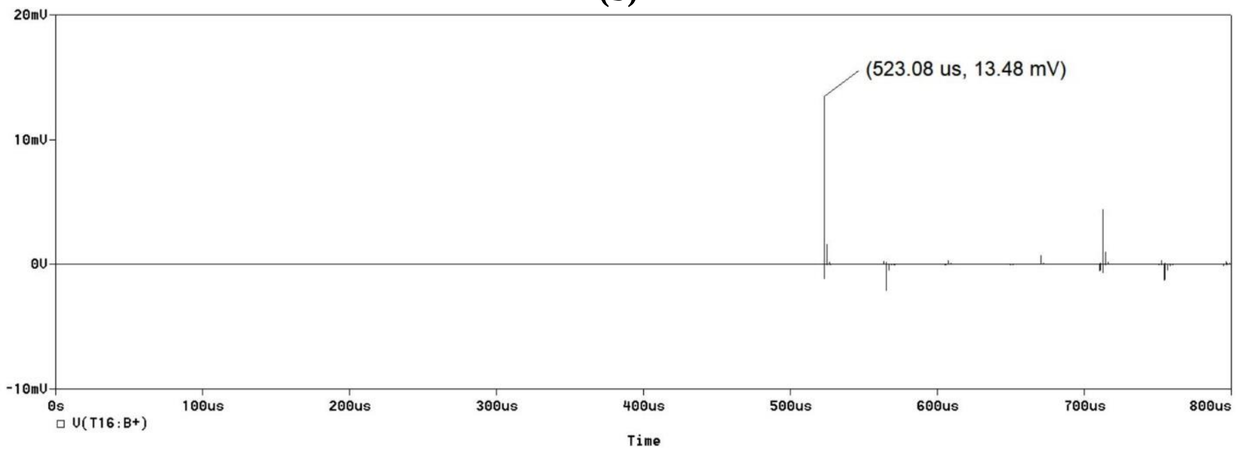

20mV
10mV
0V
-10mV
(523.08 us, 13.48 mV)
0s
100us
200us
300us
400us
500us
600us
700us
800us
V(T16:B+)
Time

(c)

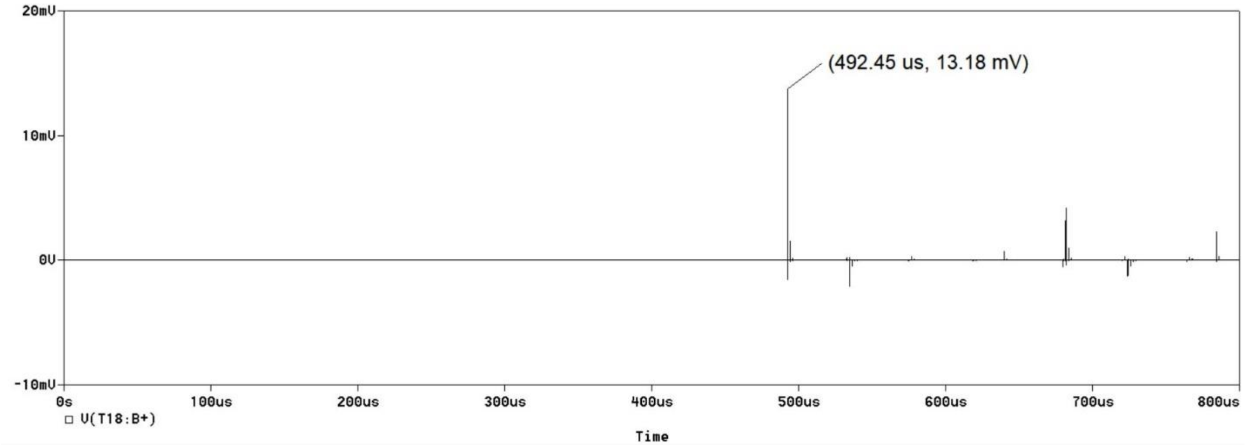

(d)

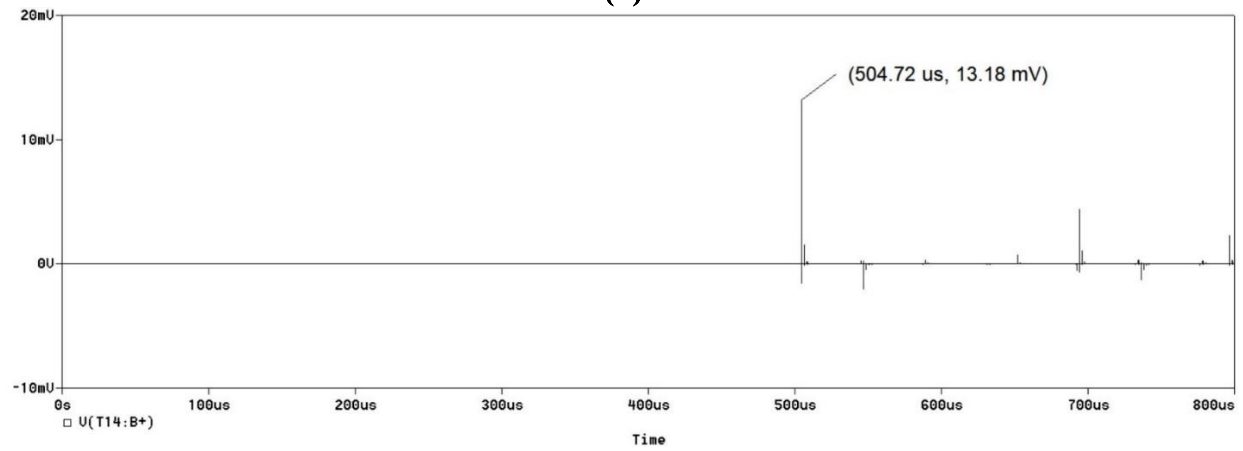

(e)

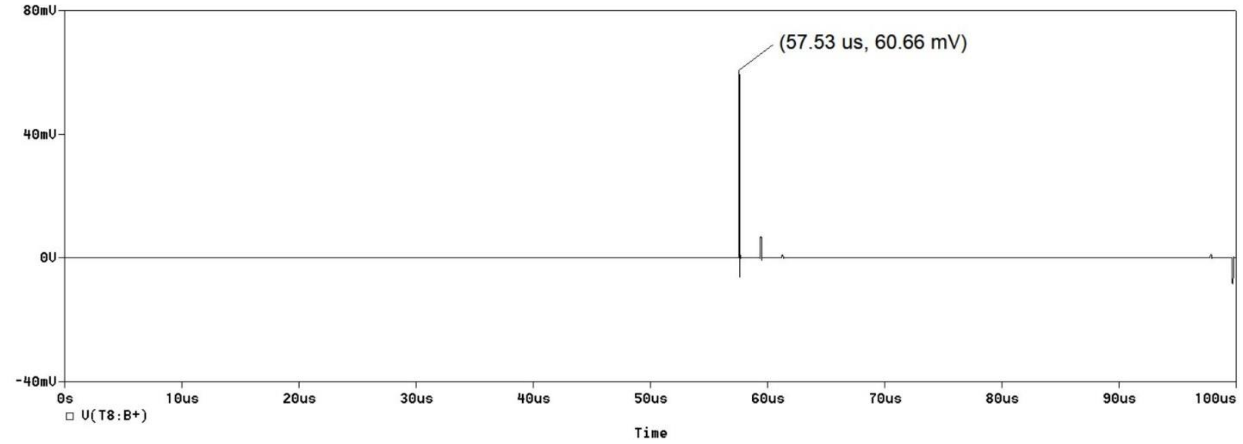

(f)

Supplementary Figure S4. A-scan signals produced in 0.83 MPa PSpice simulation. The measured voltages from the cervical simulation are seen in (a) *v*(T7:B+) for the left recurrent laryngeal nerve, (b) *v*(T9:B+) for the cardiac plexus, (c) *v*(T16:B+) for the hepatic plexus, (d) *v*(T18:B+) for the gastric plexus, and (e) *v*(T14:B+) for the splenic nerve. The measured voltage from the site-focused simulation is seen in (f) *v*(T8:B+) for the splenic nerve

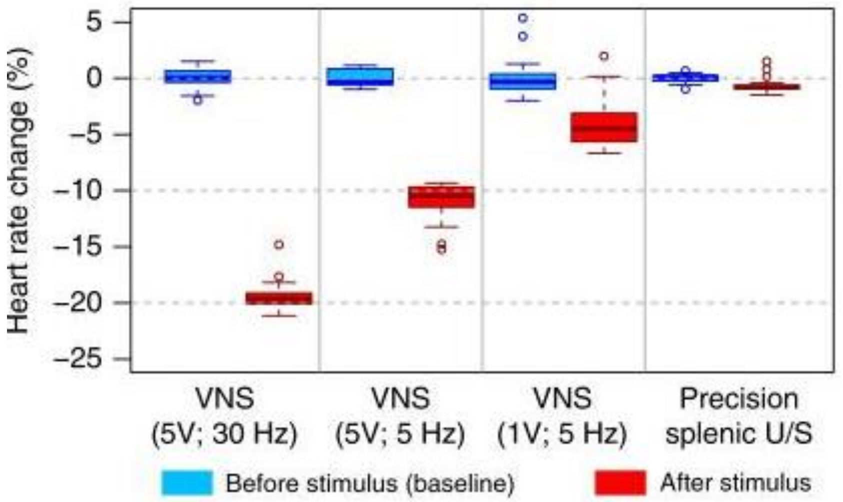

Supplementary Figure S5. Comparison of cervical vagus nerve stimulation with site-focused splenic nerve ultrasonic stimulation. Data from Cotero et al. comparing cervical vagus nerve stimulation at varying frequencies to the focused ultrasonic treatment at the spleen with regards to their effect on heart rate. The precision splenic U/S had a 1.1 MHz frequency [1]

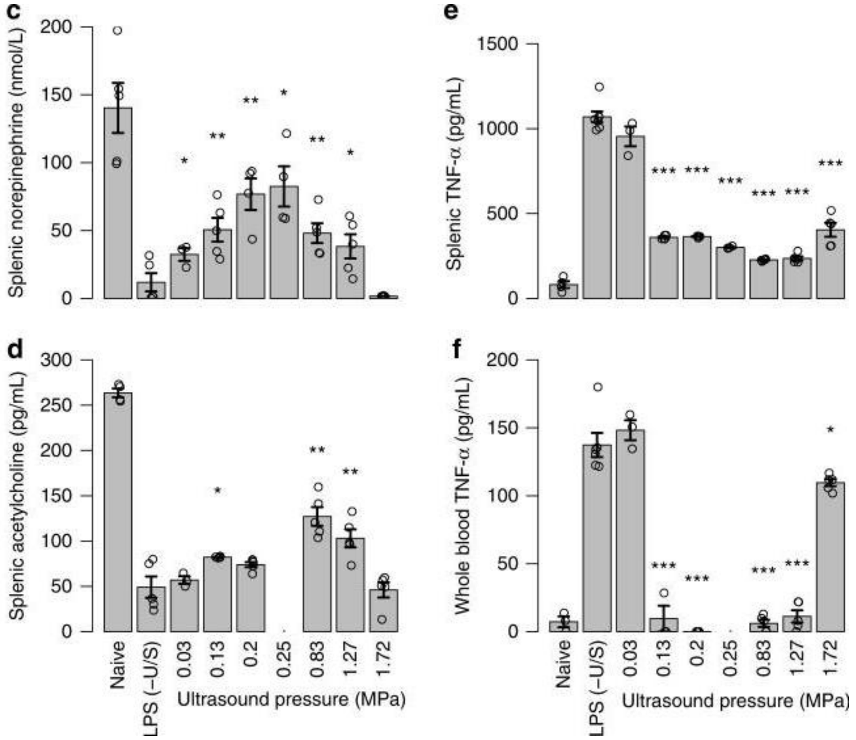

Supplementary Figure S6. Ultrasonic neurostimulation treatment on inflammatory biomarkers. Experimental results from Cotero et al. of ultrasonic neurostimulation on c) splenic norepinephrine, d) acetylcholine, e) TNF-α, and f) whole blood TNF-α [1]

[1] Cotero, V. *et al.* Noninvasive sub-organ ultrasound stimulation for targeted neuromodulation. *Nat. Commun.* **10**, 952 (2019).
